# Compensating Unknown Time-Varying Delay in Opto-Electronic Platform Tracking Servo System

**DOI:** 10.3390/s17051071

**Published:** 2017-05-09

**Authors:** Ruihong Xie, Tao Zhang, Jiaquan Li, Ming Dai

**Affiliations:** 1 Graduate University of Chinese Academy of Sciences, Beijing 100039, China; 2 Key Laboratory of Airborne Optical Imaging and Measurement, Changchun Institute of Optics, Fine Mechanics and Physics Chinese Academy of Sciences, Changchun 130033, China; zhangt@ciomp.ac.cn (T.Z.); lijiaquan163163@163.com (J.L.); daim@ciomp.ac.cn (M.D.)

**Keywords:** miss-distance, unknown time-varying delay, Markovian process, feed-forward forecasting, opto-electronic platform, tracking servo system, robust H∞ controller

## Abstract

This paper investigates the problem of compensating miss-distance delay in opto-electronic platform tracking servo system. According to the characteristic of LOS (light-of-sight) motion, we setup the Markovian process model and compensate this unknown time-varying delay by feed-forward forecasting controller based on robust H∞ control. Finally, simulation based on double closed-loop PI (Proportion Integration) control system indicates that the proposed method is effective for compensating unknown time-varying delay. Tracking experiments on the opto-electronic platform indicate that RMS (root-mean-square) error is 1.253 mrad when tracking 10° 0.2 Hz signal.

## 1. Introduction

**Figure 1:**
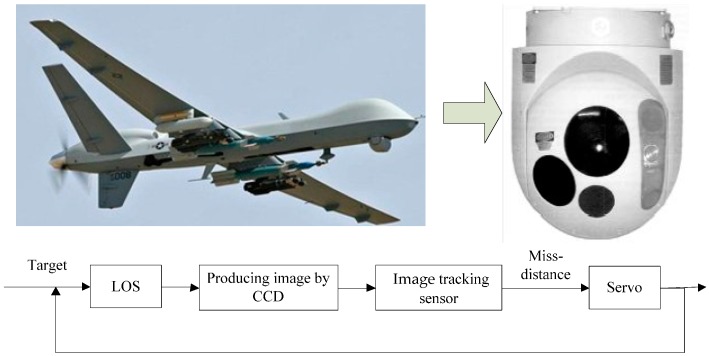
The opto-electronic platform tracking servo system.

The opto-electronic platform can quickly capture and track the moving target on the aircraft, which had a widely application in aircraft reconnaissance surveying, searching, rescuing and assessing shells-hitting result. As shown in Figure 1, miss-distance between the moving target and LOS was measured by image tracking sensor in the opto-electronic platform. Then, this miss-distance was sent to servo system and the DC motor was controlled by servo system to produce the corresponding movement for eliminating miss-distance and tracking the target.

However, there was a non-negligible miss-distance delay caused by the process of producing image by CCD (Charge Coupled Device), measuring miss-distance by image tracking sensor and transmitting miss-distance data to servo system. This miss-distance delay in the opto-electronic platform tracking servo system can be described as unknown, bounded and time-varying. It reduced the bandwidth, tracking accuracy and even caused servo system to oscillate. Consequently, compensating miss-distance delay in opto-electronic platform was absolutely necessary.

In the past decades, control systems with time-delay have attracted much attention. To the best of our knowledge, the engineering solution of this problem was roughly divided into two categories. The widely used methods are probably to design the appropriate controller *u*(*t*)*/U*(*K*) directly. As noticed in [1], time-varying delay has received very little attention. Until very recently, heavily research has done on infinite-time systems with time-varying delay [1,2,3,4,5,6,7,8,9,10,11,12]. With the development of the linear matrix inequality (LMI) approach, robust H∞ controller for time-delay systems has been greatly discussed for stochastic systems [13,14,15,16,17,18,19,20]. However, design of the controller *u*(*t*)*/U*(*K*) directly under unknown time-varying delay cannot meet the accuracy requirements because tracking accuracy must be mrad level.

Another efficient approach is compensating time-delay by feed-forward forecasting based on maneuvering target tracking [21,22,23,24,25,26,27,28], such as such as particle filter [23], Kalman filter [24] and H∞ filter [27], which was already used for compensating miss-distance delay in opto-electronic platform tracking servo system. However, all those methods can only be used for compensating constant-time delay. To the best of our knowledge, very little attention has been paid to the problem of feed-forward forecasting controller for discrete-time Markovian systems with unknown time-varying delay.

In this paper, we focus on compensating unknown time-varying delay in the opto-electronic platform tracking servo system and design a new feed-forward forecasting controller based on robust H∞ controller. Simulation based on double closed-loop PI control system indicates that the proposed method is effective for compensating unknown time-varying delay. Tracking experiments on the opto-electronic platform indicate that root-mean-square (RMS) error is 1.253 mrad when tracking 10° 0.2 Hz signal. The remainder of this paper is organized as follows. Section 2 analyzes effect of miss-distance delay on the opto-electronic platform tracking servo system. The proposed method is presented in Section 3. Section 4 presents the experiment base on double closed-loop PI control system and some conclusions of this study are given in Section 5.

## 2. Problem Statement

The most effective control program for the opto-electronic platform tracking servo system was double closed-loop control, the position control loop based on opto-electronic encoder and the velocity control loop based on rate gyro, which had been proven to be effective in numerous applications over the years [29].

**Figure 2:**
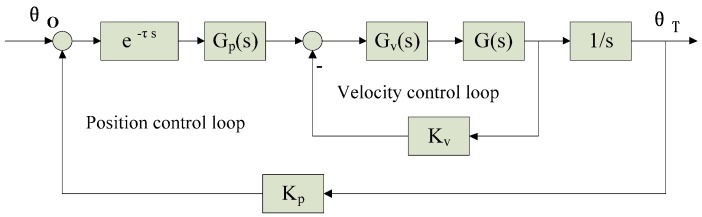
Diagram of position control loop and velocity control loop.

As shown in Figure 2, effect of miss-distance delay on the opto-electronic platform tracking servo system is equal to adding the transfer function e^−τs^ on the position control loop [30,31,32]. The frequency characteristics of e^−τs^:

(1) $$\{\begin{array}{l} A_{\tau }(jw)=1\\ \phi_{\tau }(jw)=-w\tau \end{array}$$

where *A_τ_*(*jw*) is the amplitude-frequency characteristics, and *ф_τ_*(*jw*) is the phase-frequency characteristics. Equation (1) indicates that miss-distance delay only affects the phase characteristics.

The lost phase margin:

(2) $$\phi_{l}=2\pi f_{c}d(t)$$

where *f_c_* is the crossover frequency, and *d*(*t*) is the miss-distance delay.

(3) $$G(s)=\frac{1/C_{e}}{\left(T_{m}s+1)(T_{e}s+1\right)}$$

**Figure 3:**
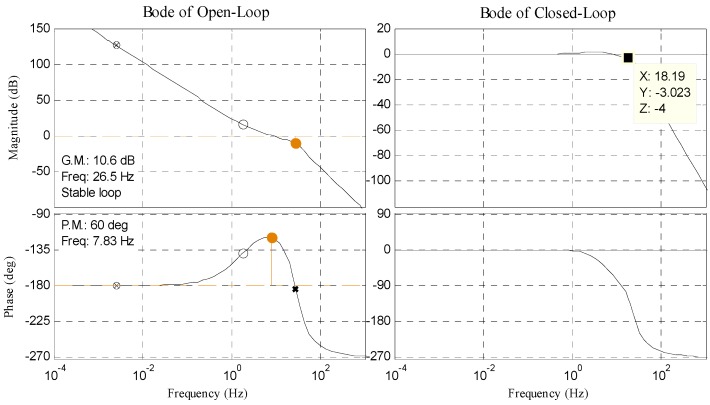
Bode diagram of open position-loop and closed position-loop in no-delay situation.

**Figure 4:**
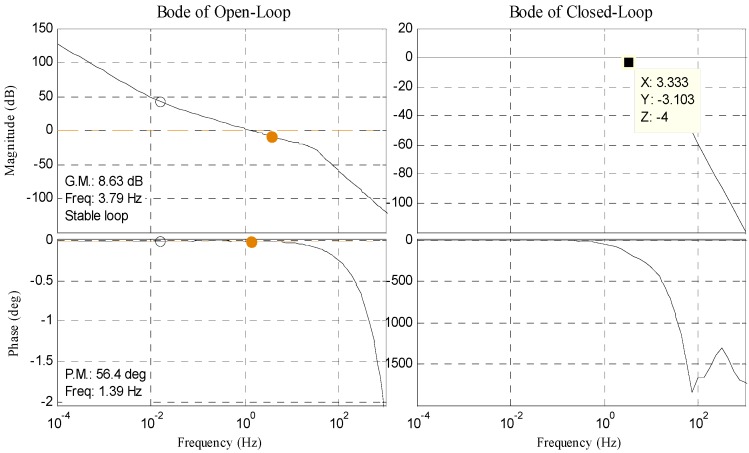
Bode diagram of open position-loop and closed position-loop in delay situation.

**Figure 5:**
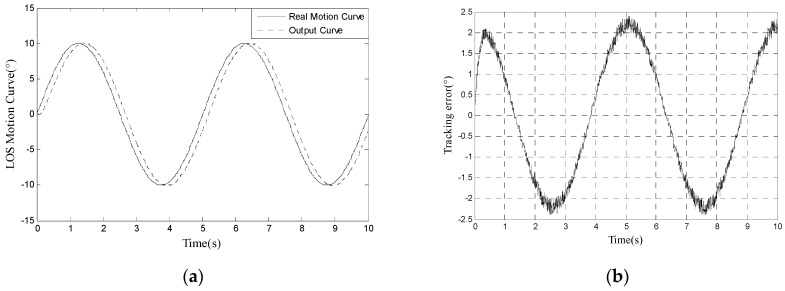
(**a**) LOS motion curve of tracking 10° 0.2 Hz signal without compensating miss-distance delay; and (**b**)tracking error of tracking 10° 0.2 Hz signal without compensating miss-distance delay.

The controlled object *G*(*s*) can be written as Equation (3), where *C_e_* is the back electromotive force of DC motor, *T_m_* is the electromechanical time, and *T_e_* is the electromagnetic time. In this paper, we separately design double closed-loop PI controller in no-delay situation and delay situation. Bode diagram of open position-loop and closed position-loop in each situation is shown in Figure 3 and Figure 4. The result of tracking 10° 0.2 Hz signal in delay situation is shown in Figure 5.

Contrasting Figure 4 with Figure 3, the bandwidth is reduced from 18 Hz to 3 Hz. As shown in Figure 5, RMS error is 1.6474° when tracking 10° 0.2 Hz signal, which cannot satisfy engineering indicator 1.5 mrad (0.0860°).

## 3. Proposed Method

### 3.1. Time-Varying Delay Model Setup

The opto-electronic platform tracking servo system contains azimuth controller and pitch controller. Considering the design of azimuth controller and pitch controller are the same, in this paper, we only design the azimuth controller. This problem can be described as the following liner discrete-time Markovian system:

(4) $$\{\begin{array}{l} X(k+1)=(A(r_{k})+\Delta A(r_{k}))X(k)+(B(r_{k})+\Delta B(r_{k}))U(k)+CW(k)\\ Y(k)=H(r_{k})X(k) \end{array}$$

(5) $$X(k)={\left[x_{1}(k)\;x_{2}(k)\;x_{3}(k)\right]}^{T}$$

where *X*(*k*) is system state vector and is shown in Equation (5), *x*_1_(*k*) is position data at time *k*, *x*_2_(*k*) is velocity data at time *k* and *x*_3_(*k*) is acceleration data at time *k*. *U*(*k*) is the mean acceleration at time *k*, *Y*(*k*) is the measured output at time *k*, and *W*(*k*) is noise matrix which belongs to l_2_[0,∞). *A*(*r_k_*)*, B*(*r_k_*)*, C,* and *H*(*r_k_*) are known matrices. ∆*A*(*r_k_*) and ∆*B*(*r_k_*) are unknown delay matrices related to the unknown time-varying delay *d*(*k*). *d*(*k*) is satisfied with:

(6) $$0\leq d_{\min}\leq d(k)\leq d_{\max}$$

Let {*r_k_*, *k* ∈ *Z*+} be discrete-time Markov process, which takes values on finite space S = {0,1,2 ,…,*N*} with transition rate matrix *Π* = {*π_ij_*, *i,j* ∈ S}, where *π_ij_* is the transition rate from *i* to *j* given by:

(7) $$P(r_{k+1}=j|r_{k}=i)=\pi_{\mathrm{ij}};\;0\leq \pi_{\mathrm{ij}}\leq 1$$

Define *r_k_* = *i,* then Equations (4) can be written as:

(8) $$\{\begin{array}{l} X(k+1)=A_{i}(k)X(k)+B_{i}(k)U(k)+CW(k)\\ Y(k)=H_{i}(k)X(k) \end{array}$$

where A_i_(*k*) = A(*r_k_*) + ∆A(*r_k_*), and B_i_(*k*) = B(*r_k_*) + ∆B(*r_k_*).

According to mean-adaptive acceleration model of maneuvering target, the continuous-time state equation can be described as:

(9) $$\left[\begin{array}{c} {\dot{x}}_{1}(t)\\ {\dot{x}}_{2}(t)\\ {\dot{x}}_{3}(t) \end{array}\right]=\left[\begin{array}{ccc} 0 & 1 & 0\\ 0 & 0 & 1\\ 0 & 0 & -\delta \end{array}\right]\left[\begin{array}{c} x_{1}(t)\\ x_{2}(t)\\ x_{3}(t) \end{array}\right]+\left[\begin{array}{c} 0\\ 0\\ \delta \end{array}\right]a(t)+\left[\begin{array}{c} 0\\ 0\\ 1 \end{array}\right]w(k)$$

where *a*(*t*) is the mean acceleration at time *t* and *δ* is the maneuvering frequency.

Define *t = T + d_i_*(*k*), and *A_i_*, *B_i_*, and *C* in discrete-time Markovian system (Equation (8)) satisfy Equations (10)–(12), respectively.

(10) $$A_{i}=\left[\begin{array}{ccc} 1 & t & (-1+\delta t+e^{-\delta t})/\delta^{2}\\ 0 & 1 & (1-e^{-\delta t})/\delta \\ 0 & 0 & e^{-\delta t} \end{array}\right]$$

(11) $$B_{i}=\left[\begin{array}{c} t^{2}/2\\ t\\ 1 \end{array}\right]-A_{i}=\left[\begin{array}{c} -t/\delta +t^{2}/2+(1-e^{-\delta t})/\delta^{2}\\ t-(1-e^{-\delta t})/\delta \\ 1-e^{-\delta t} \end{array}\right]$$

(12) $$C=\left[\begin{array}{c} 0\\ 0\\ 1 \end{array}\right]$$

Considering only position data *x*_1_(*k*) can be observed in the opto-electronic platform tracking servo system, observing matrix *H_i_* can be described as:

(13) $$H_{i}=\left[\begin{array}{ccc} 1 & 0 & 0 \end{array}\right]$$

### 3.2. Controller System Design

In this paper, we consider one opto-electronic platform tracking servo system with *d_min_ =* 40 ms and *d_max_ =* 80 ms. Assume *d_i_*(*k*) satisfies discrete-time Uniform Distribution, which means:

(14) $$\{\begin{array}{ll} d_{i}(k)=(40+i)/1000s & i\in S=\{0,1,\ldots ,40\}\\ P_{i}=1/41 & i\in S=\{0,1,\ldots ,40\} \end{array}$$

Define estimating state *Z*(*k*):

(15) $$Z(k)=L_{i}(k)X(k)$$

where *L_i_*(*k*) = [1,0,0]. Let $\hat{Z}(k)$ denote the estimate of *Z*(*k*) which is the measured output *Y*(*k*). The error *e*(*k*) can be written as:

(16) $$e(k)=\hat{Z}(k)-L_{i}X(k)$$

Equation (8) is robustly stochastically stable under the condition:

(17) $$E[\sum_{k=0}^{n}e^{T}(k)e(k)]\leq \gamma^{2}\sum_{k=0}^{n}w^{T}(k)w(k)$$

where *γ* is H∞ level. Equation (17) is satisfied with appropriate H∞ level *γ* and *i* ∈ *S,* if and only if there exist *P_i_*(*k+*1|*k*) such that following matrix inequalities hold:

(18) $${P_{i}}^{-1}(k+1|k)+{H_{i}}^{T}(k)H_{i}(k)-\gamma^{-2}{L_{i}}^{T}(k)L_{i}(k)>0$$

where *P_i_*(*k+*1|*k*)satisfies the Discrete-time Riccati Equation:

(19) $$P_{i}(k+1|k)=A_{i}P_{i}(k|k){A_{i}}^{T}+CC^{T}-A_{i}P_{i}(k|k)\left[\begin{array}{cc} {H_{i}}^{T} & {L_{i}}^{T} \end{array}\right]{R_{i}}^{-1}(k)\left[\begin{array}{c} H_{i}\\ L_{i} \end{array}\right]P_{i}(k|k){A_{i}}^{T}$$

(20) $$R_{i}(k)=\left[\begin{array}{cc} I & 0\\ 0 & -\gamma^{2}I \end{array}\right]+\left[\begin{array}{c} H_{i}\\ L_{i} \end{array}\right]P_{i}(k|k)\left[\begin{array}{cc} {H_{i}}^{T} & {L_{i}}^{T} \end{array}\right]$$

**Figure 6:**
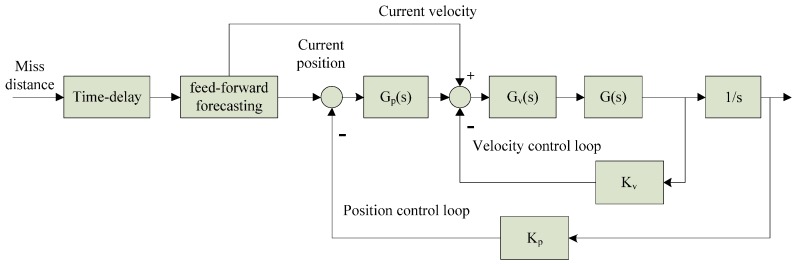
Diagram of feed-forward forecasting controller in opto-electronic platform.

According to the analysis above, feed-forward forecasting controller system is design as follows:

(1) Select minimal *γ >* 0 and *i* ∈ *S* which can satisfy Equations (18)–(20). *γ* is a constant that is selected by testing experiment to satisfy the requirement of engineering and *i* is time-varying.
(2) Prediction:
  (21) $${\hat{X}}_{i}(k+1|k)=A_{i}{\hat{X}}_{i}(k)+B_{i}U(k)$$
  (22) $$K_{i}(k+1)=P_{i}(k+1|k){H_{i}}^{T}\cdot {\left[I+H_{i}P_{i}(k+1|k){H_{i}}^{T}\right]}^{-1}$$
  where *P_i_*(*k+*1|*k*) and *R_i_*(*k*) are shown in Equations (19) and (20).
(3) Measurement update:
  (23) $${\hat{X}}_{i}(k+1)={\hat{X}}_{i}(k+1|k)+K_{i}(k+1)\cdot (Y(k+1)-H_{i}{\hat{X}}_{i}(k+1|k))$$
  (24) $$P_{i}(k+1|k+1)={\left[{P_{i}}^{-1}(k+1|k)+{H_{i}}^{T}(k)H_{i}(k)-\gamma^{-2}{L_{i}}^{T}(k)L_{i}(k)\right]}^{-1}$$
(4) Transmitting current data:
  As shown in Figure 6, we transmit the current position ${\hat{x}}_{1}(k+1)$ and velocity ${\hat{x}}_{2}(k+1)$ to position/velocity control loop separately after feed-forward forecasting. The DC motor was controlled by servo system to produce the corresponding movement for eliminating miss-distance and tracking the target.

## 4. Experiment

Considering one opto-electronic platform tracking servo system, *C_e_ =* 1.333 V, *T_m_* = 0.7 s, *T_e_* = 0.006 s, sample frequency of velocity-loop is 500 Hz, and sample frequency of position-loop is 50 Hz. According to Equation (3), controlled object *G*(*s*) can be written as:

(25) $$G(s)=\frac{0.75}{\left(0.7s+1)(0.006s+1\right)}$$

We design *G_v_*(*s*) and *G_p_*(*s*) as shown in Equation (25). The bandwidth of velocity-loop is 28 Hz. The bandwidth of position-loop is 18 Hz. Both of them satisfy engineering indicator.

(26) $$\{\begin{array}{l} G_{v}(s)=\frac{2.6584(62s+1)}{s}\\ G_{p}(s)=\frac{535.93(0.12s+1)}{s} \end{array}$$

The sample period *T_s_* is 0.02 s and *γ =* 0.8 is selected by testing experiment. The actual time from *X*(*k*) to *X*(*k+1*) can be written as:

(27) $$t=T+d_{i}(k)=(0.06+i/1000)s\;i\in S=\{0,1,\ldots ,40\}$$

**Figure 7:**
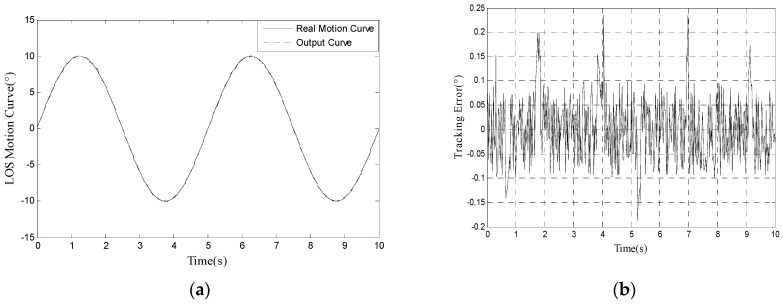
(**a**) LOS motion curve of our method when tracking 10° 0.2 Hz signal; and (**b**) tracking error of our method when tracking 10° 0.2 Hz signal.

We use the proposed method to track 10° 0.2 Hz input signal and the LOS motion curve is shown in Figure 7a. The real motion curve and the output curve are almost overlapped because the amplitude of LOS motion curve is far bigger than tracking error. The tracking error is shown in Figure 7b. In Figure 7b, we can calculate that RMS error is 0.0673°, which satisfies engineering indicator 1.5 mrad (0.0860°).

In order to verify the performance of the proposed method for compensating unknown time-varying delay, we conduct contrast experiments based on Kalman filter/H∞, filter which were used for compensating constant-delay in the opto-electronic platform tracking servo system before. Those constant time-delay methods are shown as follows:

(28) $$\{\begin{array}{l} X(k+1)=\Phi X(k)+CW(k)\\ Y(k)=HX(k)+V(k) \end{array}$$

where *W*(*k*) and *V*(*k*) are unrelated Gaussian white noise and satisfy Equations (29). Φ, *C* and *H* are known matrices which, respectively, satisfy Equations (30)–(32).

(29) $$\{\begin{array}{l} E[W_{k}]=0;\;E[W_{k}{W_{j}}^{T}]=Q_{k}\delta_{kj}\\ E[V_{k}]=0;\;E[V_{k}{V_{j}}^{T}]=R_{k}\delta_{kj}\\ E[W_{k}{V_{j}}^{T}]=0 \end{array}$$

(30) $$\Phi =\left[\begin{array}{ccc} 1 & T_{s} & {T_{s}}^{2}/2\\ 0 & 1 & T_{s}\\ 0 & 0 & 1 \end{array}\right]=\left[\begin{array}{ccc} 1 & 0.02 & 0.0002\\ 0 & 1 & 0.02\\ 0 & 0 & 1 \end{array}\right]$$

(31) $$C=\left[\begin{array}{c} 0\\ 0\\ 1 \end{array}\right]$$

(32) $$H=\left[\begin{array}{ccc} 1 & 0 & 0 \end{array}\right]$$

We also consider the opto-electronic platform tracking servo system with unknown time-varying delay, which ranges from 40 ms to 80 ms. For those constant time-delay methods, assume *d*(*t*) satisfies:

(33) $$d(t)=(d_{\min}+d_{\max})/2=60\mathrm{ms}$$

**Figure 8:**
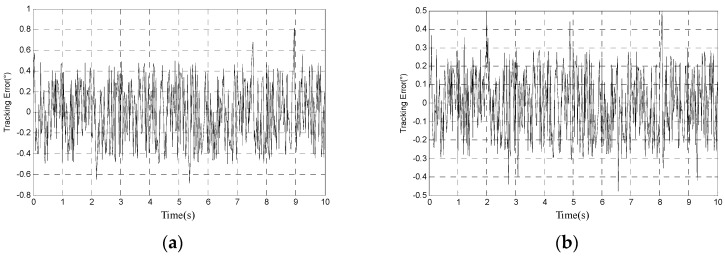
(**a**) Tracking error of Kalman filter when when tracking 10° 0.2 Hz signal; and (**b**) tracking error of H∞ filter when when tracking 10° 0.2 Hz signal.

From Equation (33) we can see that *d*(*t*) is three times the sample period *T_s_* and this 60 ms constant-delay can be compensated by three steps Kalman filter/H∞ filter. The tracking error of Kalman filter/H∞ filter for tracking 10° 0.2 Hz signal is separately shown in Figure 8a,b.

As shown in Table 1, we make a comparison of tracking accuracy according to the experiment. The RMS error of Kalman filter is 0.3022° and RMS error of H∞ filter is 0.1839°. Both of them cannot satisfy engineering indicator 1.5 mrad (0.0860°). It seems that they are unable to compensate unknown time-varying delay in opto-electronic platform tracking servo system. It also indicates that our method is effective for compensating unknown time-varying delay and satisfied engineering indicator in the opto-electronic platform tracking servo system.

**Figure 9:**
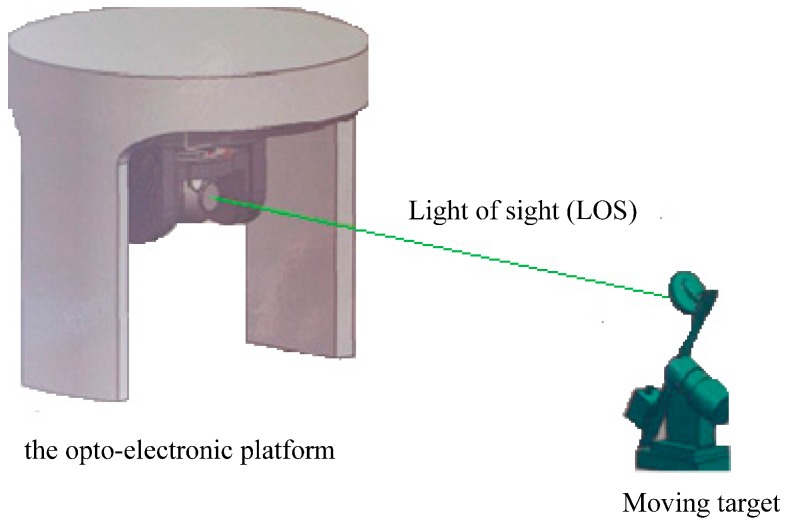
Schematic diagram of tracking experiment on the opto-electronic platform.

**Figure 10:**
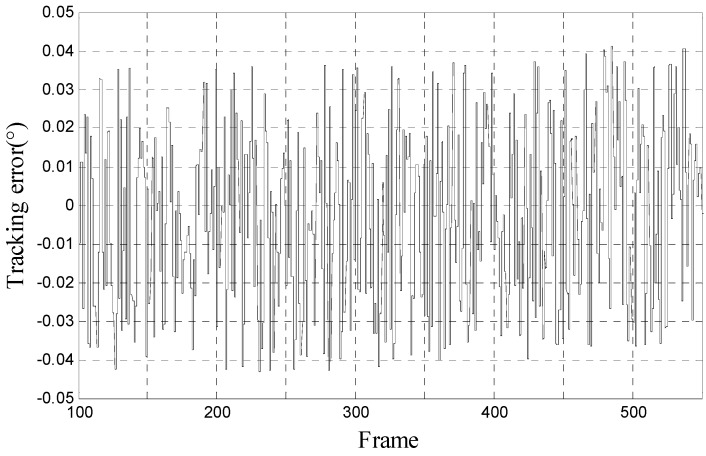
Tracking error of proposed method when moving-target move with 10° 0.05 Hz.

**Figure 11:**
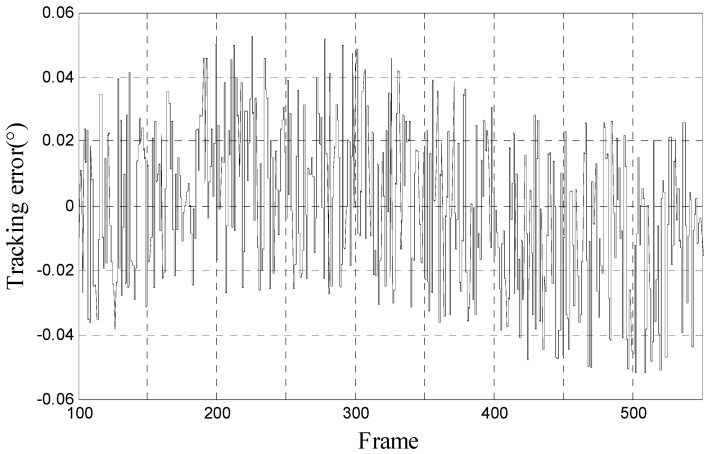
Tracking error of proposed method when moving-target move with 10° 0.1 Hz.

**Figure 12:**
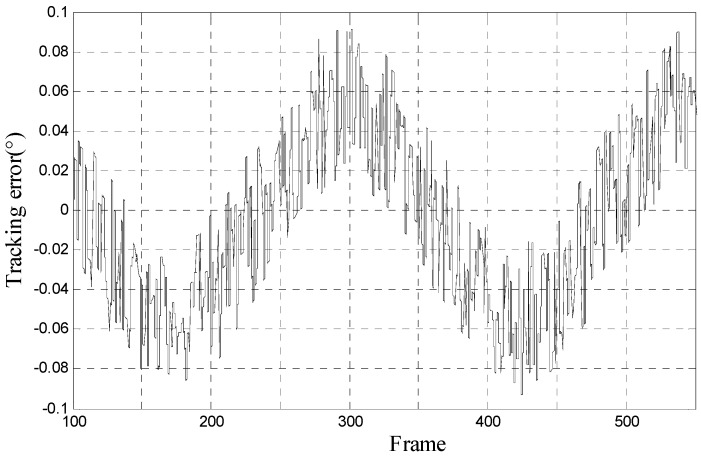
Tracking error of proposed method when moving-target move with 10° 0.2 Hz. (Note: Figure 10, Figure 11 and Figure 12 are drawn by the miss-distance data in the opto-electronic platform with 50 frames per second).

In order to verify the simulation result of proposed method above, we do tracking experiments on the opto-electronic platform. As shown in Figure 9, we fix the opto-electronic platform and make the moving-target move with 10° 0.05 Hz, 10° 0.1 Hz, and 10° 0.2 Hz, separately. We can know the tracking error by outputting the miss-distance data in the opto-electronic platform. The result of tracking error with 10° 0.05 Hz, 10° 0.1 Hz, and 10° 0.2 Hz moving-target is shown in Figure 10, Figure 11 and Figure 12, respectively.

**Figure 13:**
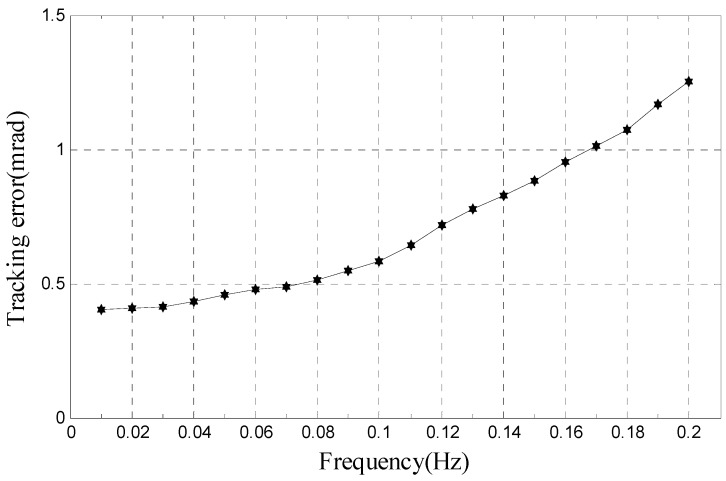
Proposed method’s relationship of tracking error and frequency.

By tracking experiments of moving target with amplitude 10° and frequency less than 0.2 Hz, we obtain the relationship of tracking error and frequency, as shown in Figure 13. It shows that tracking error is less than 1.253 mrad under the situation that amplitude is 10° and frequency is less than 0.2 Hz, which is similar to simulation result (1.175 mrad).

## 5. Conclusions

Miss-distance delay in the opto-electronic platform tracking servo system reduces the bandwidth and tracking accuracy, even causing the system to oscillate. To compensate for this unknown time-varying delay in the opto-electronic platform tracking servo system, we setup the Markovian process model and design a new feed-forward forecasting controller based on robust H∞ controller in this paper. Simulation based on double closed-loop PI control system indicates that the proposed method is effective for compensating unknown time-varying delay. The bandwidth is improved from 3 Hz to 18 Hz. Finally, tracking experiments on the opto-electronic platform indicate that root-mean-square (RMS) error is 1.253 mrad when tracking 10° 0.2 Hz signal.

## Figures and Tables

**Table 1:** Tracking accuracy comparison.

Situation/Method	RMS Error
without compensating delay	1.6474°/28.75 mrad
Kalman filter	0.3022°/5.274 mrad
H∞ filter	0.1839°/3.209 mrad
Proposed method	0.0673°/1.175 mrad

Note: Input signal of all the Situations/Methods is 10° 0.2 Hz.
